# Effect of Graphene Addition on Polycaprolactone Scaffolds Fabricated Using Melt-Electrowriting

**DOI:** 10.3390/polym14020319

**Published:** 2022-01-13

**Authors:** Johnson H. Y. Chung, Sepidar Sayyar, Gordon G. Wallace

**Affiliations:** 1 Intelligent Polymer Research Institute, Australian Research Council Centre of Excellence for Electromaterials Science, University of Wollongong, Wollongong, NSW 2500, Australia; sepidar@uow.edu.au (S.S.); gwallace@uow.edu.au (G.G.W.); 2 Australian National Fabrication Facility—Materials Node, Innovation Campus, University of Wollongong, Wollongong, NSW 2500, Australia

**Keywords:** melt-electrowriting (MEW), biofabrication, polycaprolactone, graphene composites, degradation studies

## Abstract

Melt-electrowriting (MEW) is an emerging method that combines electrospinning and extrusion printing, allowing the fabrication of micron-scale structures suitable for tissue engineering. Compared to other additive fabrication methods, melt-electro written structures can offer more appropriate substrates for cell culture due to filament size and mechanical characteristics of the fabricated scaffolds. In this study, polycaprolactone (PCL)/graphene composites were investigated for fabrication of micron-size scaffolds through MEW. It was demonstrated that the addition of graphene can considerably improve the processability of PCL to fabricate micron-scale scaffolds with enhanced resolution. The tensile strength of the scaffold prepared from PCL/graphene composite (with only 0.5 wt.% graphene) was proved significantly (by more than 270%), better than that of the pristine PCL scaffold. Furthermore, graphene was demonstrated to be a suitable material for tailoring the degradation process to avoid undesirable bulk degradation, rapid mass loss and damage to the internal matrix of the polymer. The findings of this study offer a promising route for the fabrication of high-resolution scaffolds with micron-scale resolution for tissue engineering.

## 1. Introduction

Tissue engineering is an evolving strategy for repairing damaged tissues or organs. This approach offers an efficient alternative for complicated treatments such as organ transplantation or implantation of artificial prostheses [1,2]. The manufacture of 3D scaffolds with appropriate size, shape and tissue compatibility is crucial for successful tissue regeneration. However, it is difficult to find a biocompatible polymer that meets all processing, biological and mechanical requirements.

The solution, therefore, lies in the development of biocomposites that are composed of a matrix infiltrated with a filler to compensate the deficiencies of the matrix. Among synthetic polymers, polycaprolactone (PCL) is a popular candidate for developing biocomposites. PCL is a Food and Drug Administration (FDA) approved bioresorbable polyester and has been used for medical devices since the 1980s [3]. It is one of the most commonly investigated biocompatible polymers for 3D printing because of its low melting point of 60 °C [4,5]. Carbonaceous fillers, on the other hand, have been extensively studied in the field of tissue engineering as a reinforcement to synthetic scaffolds due to their outstanding mechanical properties, ease of processing and high conductivity [6]. In particular, graphene, a unique two-dimensional carbon structure with excellent electrical, thermal and mechanical properties, is one filler that shows extensive use in the development of biocomposites [7,8,9,10,11]. The incorporation of graphene-based fillers into polymer matrices has shown improved cell adhesion and proliferation without reducing cell viability [12]. 

Processing materials into scaffolds with appropriate properties is the next challenge in the tissue engineering field. The emergence of 3D printing opened new avenues for the fabrication of complex structures for a variety of biomedical applications. Compared to conventional manufacturing methods, such as mould casting, 3D printing provides the advantage of fabricating structures with higher resolution and complexity at a faster speed. This technique can also be used to fabricate customised structures to accurately mimic the biomechanical properties of damaged organs and tissues [11,13]. In extrusion 3D printing, ink is extruded through a nozzle to produce a construct based on a 3D model. However, this technique is challenged with printing fine strut sizes (<100 μm). The strut size plays a pivotal role in the field of tissue engineering as they can influence the behaviour of various cell types [14,15]. Struts with a diameter of 10–20 μm are not only comparable with the fibrous structure of tissues but also are the most suitable size for osteogenic differentiation of cells [16]. MEW has gained increased attention over the past few years. The ability to place fibres accurately in a scale much smaller than 3D extrusion based printing systems provides numerous opportunities in scaffold fabrication and design [17,18]. By using MEW to produce micron-size PCL struts, it can not only increase the flexibility of the scaffold overall but also potentially influence the degradation rate and elicit interesting cell behaviour [19,20]. Studies conducted by Freeman et al. [21] showed the possibility to direct mesenchymal stem cell (MSC) differentiation through modulating stiffness of printed constructs. MSCs preferentially underwent osteogenesis in stiffer regions, while in softer regions they appeared to undergo equal amounts of osteogenesis and adipogenesis. 

As an implantable synthetic polymer, PCL has been studied extensively to understand the degradation mechanism and excretion pathway [4]. However, the timeframe for PCL to degrade varies from months to years. For applications such as cartilage or bone regeneration, a longer degradation may be desired, while the opposite is required for neural or other soft tissue applications. In addition, PCL is a rigid polymer that can develop sharp edges during degradation from the rapid loss of molecular weight and cause undesirable inflammation to surrounding tissues [4,22]. By printing PCL using MEW to produce thinner filament diameters, this may alter the degradation profile given the larger surface–volume ratio. The addition of graphene has been reported to impact the degradation rate while introducing additional electrical properties to PCL. Previously published studies in enzymatic degradation of graphene/PCL composites showed that the presence of reduced graphene oxide decelerated enzymatic degradation as compared to pristine PCL [8,23]. However, other in vivo studies on PCL membranes have reported reduced graphene oxide to accelerate degradation as it created higher internal porosity and facilitated water permeation [22]. In the above-mentioned studies, PCL/graphene composites were studied as a solid construct. Using MEW, the impact of graphene in PCL can be investigated as a scaffold consisting of ordered interconnected networks that may differ from the degradation mechanism observed in solid composites.

In this study, we developed micron-scale scaffolds with tailorable mechanical and degradation properties using MEW of PCL/graphene composites. We examined the effect of graphene addition on processability and thermal properties of the polymer to develop a suitable substrate for in vitro tissue models. MEW of the PCL/graphene composites was optimised to fabricate scaffolds with the finest possible strut size. The mechanical properties and degradation profile of the scaffolds were also investigated.

## 2. Materials and Methods

### 2.1. Materials

PCL (MW of 45 KDa) was obtained from Sigma Aldrich Pty Ltd (Sydney, Australia). Lipase from Pseudomonas sp. Type VIII was purchased from Sigma Aldrich. Dimethyl formamide (DMF) was purchased from Chem-Supply (Gillman, Australia).

DMF-dispersed graphene dispersion and PCL/graphene composites were prepared according to a method described in our previous work [24]. Briefly, highly reduced graphene oxide was acidified to prepare aggregated graphene powder. After neutralizing, washing and drying, the chemically converted graphene (CCG) powder was dispersed in DMF by using several cycles of sonication and centrifugation to prepare a DMF-dispersed CCG (0.5 mg/mL) dispersion that was stable for several months without any observable aggregation.

### 2.2. Preparation of PCL-CCG Composites

PCL-CCG composites with different graphene content were prepared by mixing PCL in an appropriate amount of DMF-dispersed CCG at 75 °C for 3 h. The mixture was then cooled to room temperature, precipitated in cold methanol, filtered, washed with ethanol and dried in a vacuum oven at 50 °C. The composites were labelled according to the weight percentage of the graphene content per PCL, with PCL-CCG 0.1 and PCL-CCG 0.5 containing 0.1 and 0.5 wt.% graphene, respectively. Composites with up to 10 wt.% graphene content could be prepared without observable aggregation; however, they were not processable through MEW due to increased viscosity and melting point.

### 2.3. Melt Electrowriting (MEW)

**Figure 1:**
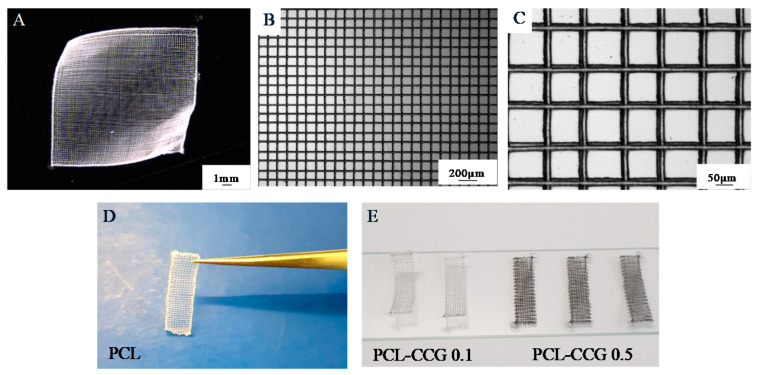
Printed scaffolds of PCL (**A**–**D**) and scaffolds to mechanical testing (**D**–**E**).

Samples were printed using a Bioscaffolder 3.2 (GeSiM®, Radeberg, Germany) fitted with an MEW module. The system is equipped with a pneumatic extrusion print head capable of performing high-voltage induced fibre deposition operating at pressures of 0–600 kPa. The positive voltage is placed on the needle tip and a negative voltage of the collecting tray. This dual voltage power supply setup is similar to methods reported in literature that increases the maximum number of layers for each construct [25]. For fabricating scaffolds, the materials (PCL or PCL composites) were loaded into stainless steel syringe barrels, kept at 100 °C for 30 min and dispensed through a 250-micrometre nozzle. Scaffolds were designed using GeSiM Robotics (1.17.4.4751) and printed at 100 °C with a strand spacing of 100 μm. In order to determine the processing parameters for obtaining a construct with the highest resolution, a range of pressures ((5–100 kPa) was tested at a constant speed of 35 mm/s and applied with 5 kV and from a distance of 4.8 mm from substrate. The minimum pressure capable to print a filament was then held constant against varying speeds of 5–55 mm/s to determine the critical translation speed necessary to print a straight and continuous filament. Scaffolds were then printed with a layer height (LH) of 10 μm, stand orientation of 0/90° for 20 layers to a final dimension of 10 × 10 × 4 mm for printability studies and 20 × 5 × 40 mm strips for mechanical testing. For degradation studies, scaffolds of 50 μm filament diameter were printed instead at a LH of 40 μm for 20 layers to a final dimension of 10 × 10 × 8 mm. Measurements and final structure of scaffolds were imaged using a Leica M205A optical microscope (Leica Microsystems, Wetzlar, Germany), as shown in Figure 1.

### 2.4. Degradation

An enzymatic degradation assay was conducted using the method published previously [26]. Lipase from Pseudomonas sp. with a specific activity of 26 U/mg was dissolved in PBS to a final activity of 4 U/mL (0.16 mg/mL). The initial weight of scaffolds was taken prior to immersing them into 2 mL of enzyme solution. Control samples were immersed in 2 mL of PBS. The tubes were incubated at 37 °C in a dry incubator for up to 96 h. The enzyme solution was replaced every 24 h, and measurements were taken after 6, 24, 48 and 96 h incubation. At every time point, samples were removed, rinsed in water, dried and weighed. The absolute average mass loss of the scaffolds was calculated, and experiments were conducted in triplicates. Statistical analysis was performed using a two-tailed *t*-test to assess the mass loss at individual time points. Significant difference was indicated by *p* < 0.05.

### 2.5. Scanning Electron Microscope (SEM) 

SEM images of printed samples were taken using a Low Vacuum Scanning Electron Microscope (LVSEM) JEOL JSM-6490LV (JEOL, Peabody, MA 01960, USA) operating in high vacuum mode. The samples were coated with 15 nm of platinum (Pt) using a Dynavac SC100MS magnetron sputter coating system (Ezzi Vision Pty Ltd., Perth, Australia). Secondary electron imaging was performed at 15 kV accelerating voltage with a probe current setting of 45 and the specimen at 20 mm working distance. Images were taken at random cross sections of the scaffolds to better image layer resolution and filament shape. Magnifications of 250×, 1500× and 2500× were taken. For degradation samples, images were taken at random locations from a top-down view at 48 h and 96 h with magnifications of 100×. A high magnification of 0.5 wt.% graphene composite at 500× and 1000× was also imaged to determine the mode of degradation.

### 2.6. Materials Characterization and Mechanical Properties

Rheological properties were measured using an AR-G2 rheometer (TA Instruments, New Castle, DE, USA) with a 12-millimetre parallel plate geometry at 100 °C, which is the processing temperature of PCL and composites. Viscosity values were measured across a shear rate range of 0.1 to 1500 1/s. The mechanical properties of the scaffolds (*n* = 5 samples per group) were measured using a Shimadzu tensile tester (EZ-L, Shimadzu, Japan). The scaffolds (20 × 5 × 40 mm) were subjected to tensile test with a constant rate to measure the maximum force (at their breakage point) and the strain, which is the percentage change in the length of the sample before it breaks. Raman spectra were recorded on a Horiba LabRam HR Evolution Raman Spectrometer using a 633 nm laser line with a 300-lines mm^−1^ grating through a 100× magnification wide angle objective. TGA was performed using a TG 209 F1 Libra (NETZSCH, Weimar, Germany) with a ramp rate of 5 °C/min up to 900 °C under a nitrogen atmosphere. Differential Scanning Calorimetry (DSC) analysis was performed by using a DSC 204 F1 Phoenix (NETZSCH, Weimar, Germany) in two cycles at 5 °C/min under a nitrogen atmosphere. The sample presealed in an aluminium pan was first heated to 110 °C (above the melting point of the polymer) and then cooled to −10 °C to record melting and crystallization peaks. 

## 3. Results and Discussions

### 3.1. Materials Characterization

#### 3.1.1. Rheology

**Figure 2:**
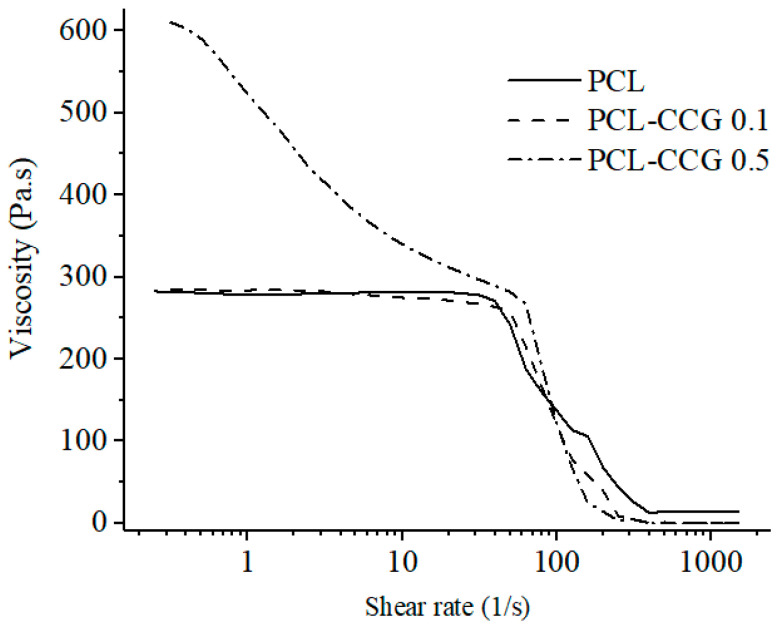
The apparent viscosity value of the PCL and PCL composites as a function of shear rate.

PCL and PCL-CCG 0.1 demonstrated three distinct first Newtonian, shear-thinning and second Newtonian regions, which is a typical viscosity curve for polymers with entangled macromolecules (Figure 2). Addition of 0.5 wt.% graphene significantly increased the viscosity of PCL from around 300 Pa·s to around 600 Pa·s. This can be attributed to the physical interactions between graphene sheets and the polymer chains. As opposed to the neat PCL and PCL-CCG 0.1, the composite with 0.5 wt.% graphene showed a profound shear thinning behaviour started from low shear rates. A broad shear thinning region is desirable in additive manufacturing as it ensures uniform material extrusion and high print fidelity. Under the shear force, graphene sheets become oriented and induce disentanglement of polymer chains that results in a gradual viscosity reduction and broad shear thinning region. By increasing the shear rate, the viscosity further reduced in all samples until it became shear rate independent, showing Newtonian behaviour.

**Figure 3:**
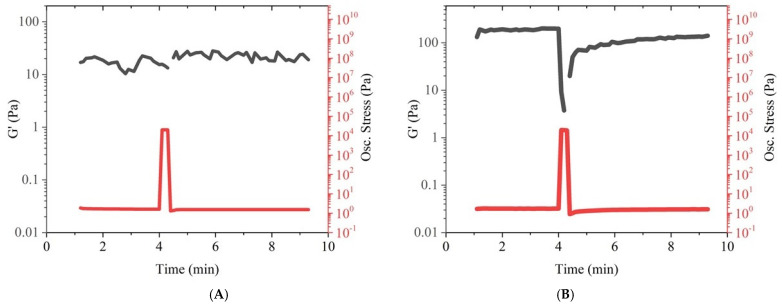
Recovery behaviour of PCL (**A**) and PCL-CCG 0.5 (**B**) under stress.

Extrusion systems subject the printing material to a high shear stress that can result in unrecoverable deformation. The ideal printing material should have a high degree of recovery after extrusion; otherwise, the formation of structures will not be possible. In order to investigate the post-printing recovery behaviour of the material, the samples were stressed at 20 kPa (above their yield point), and their recovery behaviour was investigated, as shown in Figure 3. The storage modulus (G′) represents the elastic behaviour of the material that is a crucial parameter to ensure stability of multilayer structures in additive fabrication [27]. The PCL-CCG composite showed a G′ value significantly higher than the neat PCL (100 and 10 Pa respectively), indicating much higher elastic behaviour in the composite due to the addition of graphene, which could ensure shape fidelity of the melt-electrowritten structure. The G′ value in PCL barely changed under stress due to poor elasticity of the polymer at 100 °C (Figure 3A). However, a sharp decrease followed by a fast recovery was observed in G′ of the composite sample (Figure 3B), which is a favourable behaviour in additive fabrication. During printing and under extrusion stress, elasticity of the composite reduced, which would ensure facile and consistent extrusion. The material then rapidly recovered its elasticity to retain the structure of the scaffold with high accuracy. Higher elasticity and recoverability in the composite can be attributed to graphene addition. The rigid graphene sheets form a reversible network with the polymer chains to prevent their disentanglement and slippage, enhancing elastic recovery [27,28].

#### 3.1.2. Material Characterization Composition

**Figure 4:**
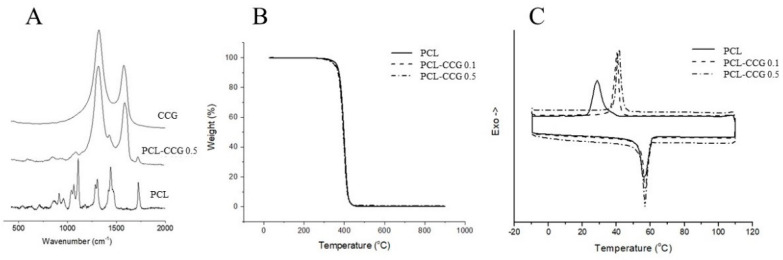
Raman spectra (**A**), TGA (**B**) and DSC (**C**) curves of PCL and PCL-CCG composites.

Raman spectra (Figure 4A) were collected between 400 and 2000 cm^−1^. The Raman spectrum of PCL shows the characteristic peaks of the polymer at 1060 cm^−1^ and 1106 cm^−1^ (skeletal vibration), 1281–1305 cm^−1^ (CH_2_ groups) and 1726 cm^−1^ (ν C=O stretching mode). The other peaks at 1470–1418 cm^−1^ (δCH_2_) and 912 cm^−1^ (νC–COO) are attributed to crystalline domains [29,30]. In samples containing graphene, two significant peaks at 1328 and 1598 cm^−1^ are observed corresponding to D and G bands of graphene sheets. The peaks due to PCL are less visible in the Raman spectra of composites as the intensity of the characteristic D and G bands of graphene are greater than that of PCL. No significant change was observed in the position or ratio of D and G bands of graphene, indicating that the structure of graphene has barely been changed when mixed with PCL. 

TGA (Figure 4B) curves demonstrate the thermal behaviour of the materials. All samples show thermal stability up to 300 °C followed by rapid decomposition due to degradation of the polymer. Graphene addition barely affected the thermal behaviour of PCL. The decomposition temperatures of PCL and PCL composites are well beyond the MEW processing temperature (100 °C), ensuring that no decomposition occurs during MEW. The residual weight after decomposition of the polymer can be assigned to graphene content as CCG weight losses are minimal in this temperature range [24]. The graphene percentage calculated from TGA analysis (*n* = 5) of the composites is consistent with the percentage of graphene added to the reaction initially, indicating homogeneous dispersion of graphene sheets in the polymer matrix.

DSC results (Figure 4C) showed that the addition of graphene up to 0.5 wt.% did not significantly affect the melting point of PCL (the melting point remains at 56–57 °C in all three samples), which is beneficial as high melting temperatures would negatively affect consistent extrusion of the material. However, the crystallization temperature of the composites is slightly higher than the pristine PCL and appeared as a sharp peak, which can be due to the nucleating effect of graphene that facilitated PCL crystallization. The results further highlight the merits of graphene addition in improving fabrication fidelity.

### 3.2. MEW of PCL Composites

MEW is a solvent-free technique that combines the principles of melt extrusion printing and electrospinning to produce polymeric highly ordered porous structures [31]. The introduction of high voltage at the nozzle causes the polymer to become charged and elongate into an electrostatically drawn jet with filament diameters typically in the range of 2–25 μm [18]. The most significant material-based parameter in MEW is the MW of the polymer. Studies have shown that as viscosity of a polymer increases, resistance to melt through the nozzle increases, thereby limiting the ability to extrude. The temperature also affects the polymer’s ability to elongate in response to increasing temperatures [32]. 

**Figure 5:**
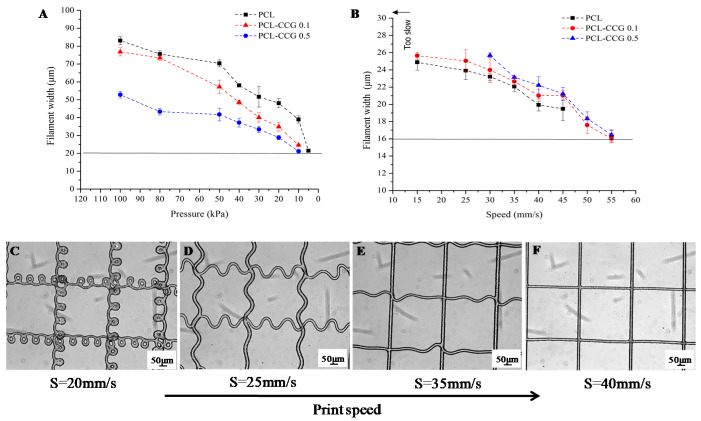
Filament width of MEW PCL against (**A**) pressure and (**B**) speed. As printing speed increases (**C**–**F**), the coiling of fibres reduced until a critical speed of 40 mm/s is reached.

Once MW and processing temperatures are established, instrument-based parameters such as voltage, distance, pressure and speed becomes important in controlling the fibre’s diameter. In this study, PCL and composites were printed at 100 °C (max. temperature), voltage of 5 kV and collector distance of 4.8 mm. The selection of a low voltage and low distance is consistent with the findings by Brown et al [33], demonstrating that the combination of low flow rate (or pressure), low collector distance and low applied voltage can result in the lowest fibre diameter. Voltages > 12 kV resulted in broken PCL fibres due to extensive drawing force, while voltages < 4 kV were insufficient to establish a Taylor cone [34]. In order to determine printing parameters to achieve the smallest filament diameter, an arbitrary speed of 35 mm/s was held constant against pressure to identify the lowest pressure to successfully extrude filaments (Figure 5A). Using optical microscopy, measurements were taken on the second layer of every print and results showed that filament width decreased with decreasing pressure across all three sample types. 

PCL was able to be extruded at 5 kPa, while composite materials require a minimum of 10 kPa due to the higher viscosity. The minimum pressure determined for all samples were then held constant against a range of printing speeds to determine the critical translation speed (CTS) necessary to print a continuous filament. Study conducted by Brown et al. [34] observed that as the melt jet impacts a stationary flat collector, it will buckle under longitudinal compression, and the fibres will coil. The frequency of the coil will be reduced by moving the collector above a critical speed so that it is moving faster than the polymer jet being deposited and sub-micrometre filaments can be drawn [25,35]. The same principle applies when the MEW print head is moving and the collector remains stationary, which is the current instrument configuration in this study. The minimum printing speed for PCL and PCL-CCG 0.1 without significant coiling is 15 mm/s, while PCL-CCG 0.5 is at 30 mm/s due to the higher viscosity. As printing speed increases, the coiling of fibres reduced until a critical speed of 40 mm/s is reached, as shown in Figure 5C–F. The thinnest filaments for all three materials were around 16 μm printed at 55 mm/s. The printing parameters for PCL, PCL-CCG 0.1 and PCL-CCG 0.5 in providing the minimum filament thickness are shown in Table 1.

**Figure 6:**
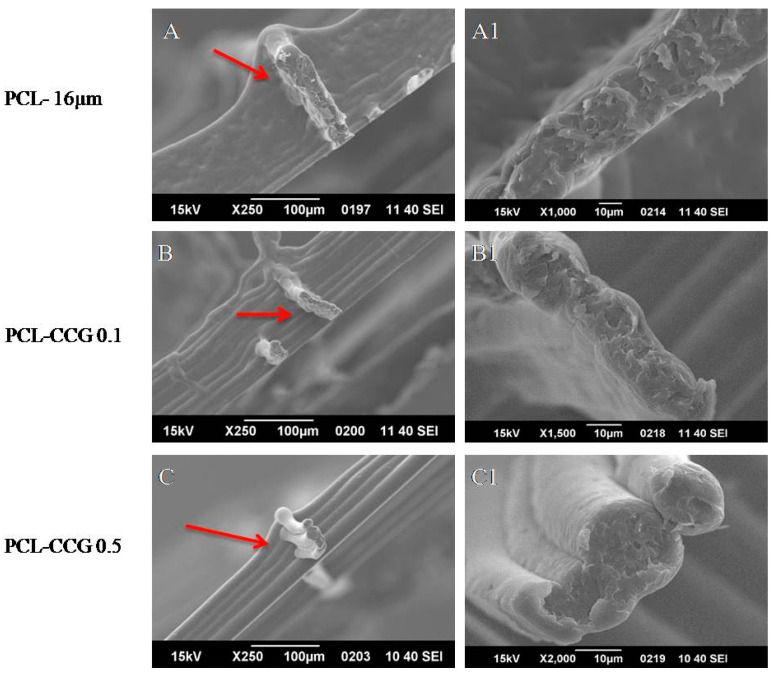
SEM images of MEW PCL and PCL composites at low magnifications (**A**–**C**) and high magnifications (**A1**–**C1**) with arrows indicating layer separation.

SEM was conducted on printed scaffolds (Figure 6). Interestingly, there was no distinct layer formation for PCL structures possibly due to its lower viscosity and storage modulus. As graphene content increased to 0.1 wt.% and 0.5 wt.%, the layer resolutions improved, and distinct layer formation was observed. Higher magnification images of PCL-CCG 0.5 (Figure 6C1) showed distinct circular fibres with no visible aggregations. The merging layers in PCL may be due to its low viscosity and storage modulus. As the viscosity of PCL at its melting point is lower than PCL-CCG 0.1 and PCL-CCG 0.5, it will flow more readily at the same temperature as the composites experience, resulting in more material deposition per second. The addition of graphene could resolve this issue by increasing viscosity in composite samples, particularly in PCL-CCG 0.5. Furthermore, due to higher storage modulus and elastic recovery in graphene containing samples, the struts could retain their shape to avoid layer diffusion after deposition. Higher crystallisation temperature in PCL-CCG composites, as evident in DSC results, further ensured faster solidification of the struts, which resulted in less shape deformation. The high print speed also does not allow layers to sufficient cool down before the next layer is deposited on top. Adding in dwell time between each layer is a potential resolution to this.

#### Structure Characterization

**Figure 7:**
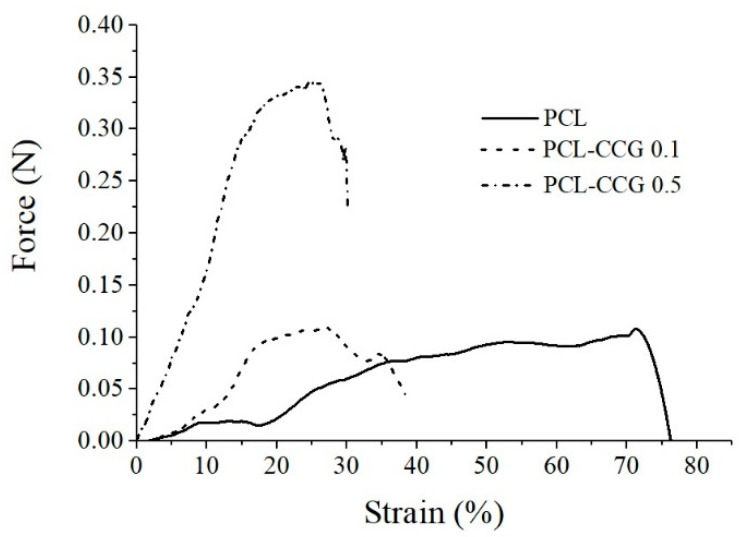
Stress–strain curves of PCL and PCL composite scaffolds.

Figure 7 compares force/strain curves of MEW PCL scaffolds (16 µm) containing 0, 0.1 and 0.5 wt.% graphene. The addition of 0.5 wt.% graphene significantly increased the strength of the PCL scaffold by more than 270% from ~0.09 N in pristine PCL to ~0.34 N in PCL-CCG 0.5 composite. The composites showed lower elongation at break (~26% strain) compared to the scaffolded fabricated using neat PCL (~70% strain), which is expected as graphene restricts the movement of polymer chains. The significant improvement in strength of the PCL-CCG 0.5 scaffold is attributed to good dispersion of graphene sheets in the polymer host and strong interaction between graphene sheets and polymer chains. 

### 3.3. In Vitro Degradation

**Figure 8:**
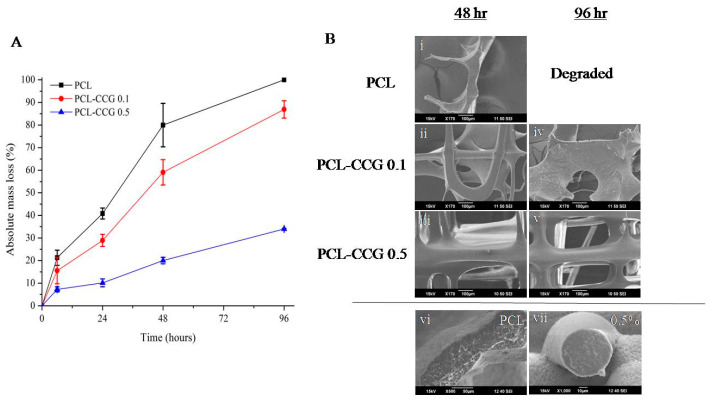
Mass loss study of MEW PCL scaffolds via enzymatic degradation (**A**); SEM images of scaffolds after enzymatic degradation (**B**) at 48 h (Bi–iii) and 96 h (Biv,v). High magnification cross-sectional images of PCL (Bvi) and PCL-CCG 0.5 (Bvii).

PCL is a semi-crystalline, hydrophobic aliphatic polyester that is currently used for sutures, wound dressing and is a common scaffold material for bone-related applications. As a long term stable polymer, PCL subjected to hydrolytic degradation requires 2–4 years for complete degradation depending on molecular weight (MW) [5]. In vivo degradation of PCL scaffolds after 6 months often shows good biocompatibility, minimal mass loss and no adverse host tissue reactions [26,36]. In this study, an accelerated degradation assay was performed on MEW-fabricated scaffolds using an enzyme. Although long-term hydrolytic studies can simulate physiological conditions better, an accelerated degradation assay can quickly assess the impact of graphene on the degradation rate and mechanism, which would otherwise take years to completely degrade [26,36]. The study was conducted using scaffolds with filament thickness of 50 μm, as the thinner strut size of 16 μm completely degraded in less than 6 h. Pristine PCL scaffolds showed the highest mass loss across samples with the initial 6 h reaching 20% compared to PCL CCG-0.1 and PCL CCG-0.5 of 15.5% and 7.3%, respectively (Figure 8A). The mass loss for PCL doubled in 24 h and again doubled at 48 h until completely dissolved. In comparison, the addition of graphene into PCL slowed the rate of degradation with PCL-CCG 0.1, but still lost ~86% after 96 h. PCL CCG-0.5 showed significantly less mass loss of only 34% after 96 h. These findings are consistent with studies conducted by Murray et al. [23], which showed that reduced graphene oxide >5 wt. % has slowed down enzymatic degradation due to higher hydrophobicity. Other studies looking at rendering the surface of FDM (fused-deposition melting)-fabricated PCL scaffolds treated with NaOH to become more hydrophilic for cell attachment also found that mass loss was 3× higher for treated scaffolds compared to untreated over 6 months [36]. Bolgen et al. [37] on the other hand demonstrated this by comparing electrospun with solvent casted PCL. By using contact angle measurements, electrospun PCLs were more hydrophobic as a result of the fibre-forming process that changed surface morphology, resulting in slower water penetration rates.

In the current study, MEW-fabricated scaffolds showed continued degradation beyond 24 h, as indicated in Figure 8A. However, there were no minor holes and pits on the filaments from SEM images (Figure 8B) for both PCL and composites. Scaffolds rapidly degrade over time and only fragments can be found for pristine PCL scaffolds as compared to composites, which still has the majority of scaffold still intact. Previous work conducted on extrusion-printed PCL noted that the porous configuration in printed scaffolds allows enzymes to penetrate into the bulk of the scaffold, steadily increasing mass loss beyond 24 h [26]. Due to the interpenetrating network of the scaffold, surface degradation occurred at the surface of the filaments but uniformly throughout the scaffold at the absence of sharp edges or weak spots. As these filaments are much thinner (50 μm) than extrusion-printed filaments (~400 μm) in earlier studies [26], it was possible that degradation occurs even faster with insufficient surface area for holes to form. This was also demonstrated in an in vitro degradation study conducted using 3D-printed PCL scaffolds [38]. It was reported that the degradation rate decreases with increasing strand diameter and porosity. This was attributed to a “wall effect” because scaffolds with larger filament diameter possess thicker walls and smaller surface area, which decreases the diffusion of degradation products and, hence, the acid-catalysed hydrolysis [39]. 

Hydrolytic degradation can proceed via surface or bulk-degradation with the former allowing predictability of the erosion process and the latter often resulting in instantaneous failure [36]. Typical surface degradation mechanisms result in thinning of the polymer over time without affecting the molecular weight or internal bulk of the polymer. The rate of hydrolytic chain scission is faster than the rate of water intrusion [40]. On the other hand, bulk degradation occurs when water is able to enter the polymer bulk, resulting in hydrolysis throughout the entire matrix. Random chain scission would take place, and the overall molecular weight of the polymer is reduced. Shorter fragments presented with carboxyl-end groups lowers pH and catalyses the degradation rate further; this is referred to as “autocatalysis”. PCL degraded via this mode is characterised with weak and hollow morphology [41]. In addition, carboxylic acid and oligomers formed during the process can result in adverse tissue reaction and inflammation [42]. Upon examining the cross section of filaments (Figure 8B), there was no evidence of bulk degradation from the cross-sectional images. These findings suggested MEW-fabricated PCL scaffolds degrade via surface erosion with the addition of graphene further altering the degradation rate, providing better control and predictability.

## 4. Conclusions

In this study, MEW of PCL graphene composites was investigated for the first time. The PCL-CCG composite was shown to be a better material for melt-electro writing of high resolution, multi-layer scaffolds, as shown by circular filaments and distinct layer separation. As confirmed by rheology and DSC results, the addition of graphene can increase the elasticity of the polymer and help it solidify faster, which will play an important role in the formation of multilayer structures with well-defined layers. Furthermore, the addition of as low as 0.5 wt.% graphene could significantly improve the strength of fabricated scaffolds by more than 270%. Bulk degradation and rapid mass loss in PCL can result in undesirable scaffold integrity and failure of implant. SEM and in vitro degradation results indicated that filaments have become thinner over time without formation of hollow filaments or holes in the internal matrix of the polymer. The degradation mechanism was evident to occur through surface erosion and uniformly through the interconnected networks and scaffold design. Degradation profiles can be altered through graphene concentration as PCL-CCG 0.5 significantly decreased the rate of degradation and extended the time. Due to increased viscosity and limitations of the instrument in extruding the composites, PCL-CCG with higher than 0.5 wt.% graphene content could not be processed, which will be the subject of future investigations.

## Figures and Tables

**Table 1:** Printing parameters for scaffolds.

Sample	Speed (mm/s)	Pressure (kPa)	Voltage (kV)	CD (mm)	Average Width (µm)
PCL	55	5	5	5.0	16.3 ± 0.6
PCL-CCG 0.1	55	10	5	4.8	16.01 ± 0.5
PCL-CCG 0.5	55	10	5	4.8	16.5 ± 0.6

## Data Availability

Not applicable.
